# Nutritional Support for Athletic Performance

**DOI:** 10.1007/s40279-015-0402-z

**Published:** 2015-11-09

**Authors:** Lawrence L. Spriet

**Affiliations:** Department of Human Health and Nutritional Sciences, University of Guelph, Guelph, ON N1G 2W1 Canada

In recent years, all personnel involved in maximizing athletic performance have adopted a science-based approach to the nutritional support of athletes and active individuals. The field of sports nutrition has exploded in terms of applied research interest and publications, and also in the practical application of the research findings to the ‘real’ or ‘athletic’ world. Many of us tend to concentrate on nutrition for hydrating and fuelling the athlete during training sessions and competitions, but we realize that the ‘before, during and after exercise’ nutritional approach is also important for recovery and preparation for the next exercise session. In addition, nutrition can also play a role in circumstances that the athlete may find themselves in, such as having to lose weight while training, recovering from an injury and trying to train part-time or full-time, and deciding whether to take nutritional supplements, such as beta-alanine or sodium bicarbonate, which would be consumed in larger quantities than found in the diet. This supplement examines many of these issues.

Applied researchers and sports personnel are continually learning more about the importance of nutrition for elite athletes. This issue considers the unequivocal importance of dietary and supplemental carbohydrate as the key fuel for success of elite-level endurance athletes, who work at extremely high exercise intensities. Elite team sports athletes also rely heavily on carbohydrate as a fuel, both for high-intensity aerobic-based activity and for sprint movements. Carbohydrate is the only fuel that can provide a substrate for both of these types of activities. Another advantage of carbohydrate is that it can be ingested during exercise and taken up and used by the skeletal muscles and central nervous system. During prolonged exercise at lower intensities, the provision of fat in the form of free fatty acids from adipose tissue can make a significant contribution to the contracting muscle’s fuel supply, but fat is not a high-intensity fuel. Not surprisingly, attempts to employ high-fat, low-carbohydrate diets to maximize elite athletic performance have not been successful. Fluid ingestion during exercise is also important, as body mass losses of ~2–3 % for endurance events and ~2 % for stop-and-go, decision-making sports can negatively affect elite performance. Typically, mass endurance events provide carbohydrate in liquid, gel and solid forms for participants, as well as various forms of liquids to maintain hydration.

The decision on whether to engage in what we might call ‘supplemental’ sports nutrition is always something to be considered for athletes. This often involves ingesting naturally occurring substances in large quantities, such as beta-alanine to increase skeletal muscle carnosine levels and intracellular buffering capacity, or sodium bicarbonate to enhance extracellular buffering capacity. In sports that require repeated sprints and generate high levels of acidity in the muscle and blood, these nutritional supplements can improve performance in many individuals.

It is also clear that nutritional support is critical in situations where athletes are attempting to lose body mass while continuing to train and prepare for competitions. Evidence suggests that lean mass can be preserved during weight loss with a sound nutritional programme. Equally important is dealing with injuries that partially or totally restrict athletes from training for varying periods of time. Nutrition can again play a major role in stimulating muscle protein synthesis and/or reducing protein breakdown during the periods of partial or total exercise restriction to counteract lean body mass losses.

The Gatorade Sports Science Institute (GSSI) brought together researchers for a meeting in March 2014 to discuss these varied topics of interest to the sports nutrition world. Following the meeting, authors were asked to summarize the recent work in their topic area, resulting in the manuscripts in this *Sports Medicine* supplement.

Lawrence L. Spriet, PhD

Guest Editor

